# Human NK Cells Lyse Th2-Polarizing Dendritic Cells via NKp30 and DNAM-1

**DOI:** 10.4049/jimmunol.1800475

**Published:** 2018-08-17

**Authors:** Katherine Walwyn-Brown, Karolin Guldevall, Mezida Saeed, Daniela Pende, Björn Önfelt, Andrew S. MacDonald, Daniel M. Davis

**Affiliations:** * Manchester Collaborative Centre for Inflammation Research, Faculty of Biology, Medicine and Health, University of Manchester, Manchester M13 9NT, United Kingdom;; † Department of Applied Physics, Science for Life Laboratory, KTH Royal Institute of Technology, Solna, SE-106 91 Stockholm, Sweden;; ‡ Laboratorio Immunologia, Istituto di Ricovero e Cura a Carattere Scientifico, Ospedale Policlinico San Martino, 16132 Genova, Italy; and; § Department of Microbiology, Tumor and Cell Biology, Karolinska Institute, 171 77 Stockholm, Sweden

## Abstract

Cross-talk between NK cells and dendritic cells (DCs) is important in Th1 immune responses, including antitumor immunity and responses to infections. DCs also play a crucial role in polarizing Th2 immunity, but the impact of NK cell–DC interactions in this context remains unknown. In this study, we stimulated human monocyte-derived DCs in vitro with different pathogen-associated molecules: LPS or polyinosinic–polycytidylic acid, which polarize a Th1 response, or soluble egg Ag from the helminth worm *Schistosoma mansoni*, a potent Th2-inducing Ag. Th2-polarizing DCs were functionally distinguishable from Th1-polarizing DCs, and both showed distinct morphology and dynamics from immature DCs. We then assessed the outcome of autologous NK cells interacting with these differently stimulated DCs. Confocal microscopy showed polarization of the NK cell microtubule organizing center and accumulation of LFA-1 at contacts between NK cells and immature or Th2-polarizing DCs but not Th1-polarizing DCs, indicative of the assembly of an activating immune synapse. Autologous NK cells lysed immature DCs but not DCs treated with LPS or polyinosinic–polycytidylic acid as reported previously. In this study, we demonstrated that NK cells also degranulated in the presence of Th2-polarizing DCs. Moreover, time-lapse live-cell microscopy showed that DCs that had internalized fluorescently labeled soluble egg Ag were efficiently lysed. Ab blockade of NK cell–activating receptors NKp30 or DNAM-1 abrogated NK cell lysis of Th2-polarizing DCs. Thus, these data indicate a previously unrecognized role of NK cell cytotoxicity and NK cell–activating receptors NKp30 and DNAM-1 in restricting the pool of DCs involved in Th2 immune responses.

## Introduction

Natural killer cells and dendritic cells (DCs) are important effectors of innate immune responses as well as priming adaptive immunity. NK cells detect stressed, infected, and tumor-transformed cells via a combination of missing self and activating receptor recognition (1, 2). They can directly lyse target cells via the secretion of cytolytic granules (3, 4) and secrete proinflammatory cytokines, which can further enhance both innate and adaptive immune responses (5–7). DCs detect infection and uptake Ags via recognition of pathogen-associated molecular patterns (8). Depending on the pathogens encountered, DCs stimulate T cells toward different types of adaptive immune responses, including Th1, Th2, and Th17 (9).

The interaction between NK cells and DCs is bidirectional (10–13). Mature DCs enhance NK cell activity via IL-15 transpresentation and secretion of IL-12 and IL-18 (14–17). In turn, NK cells aid the maturation of DCs in the presence of bacterial stimuli and augment their ability to initiate Th1 responses from naive CD4^+^ T cells (12). NK cells can lyse autologous immature DCs (12, 18) in a process involving activating receptors NKp30 and DNAM-1 (19). However, at low NK:DC ratios, secretion of IFN-γ and TNF-α can also stimulate DC maturation (20, 21). Functionally, depletion of NK cells in mice severely impacts the ability of DCs to mount a response during bacterial infection (22). Interactions between NK cells and DCs can also enhance the T cell response to cancer cells; NK cells can recruit DCs to the site of tumors, enhancing antitumor immunity (23). NK cells activated by tumor cells expressing low levels of MHC class I protein also stimulate DCs to produce IL-12 and enhance T cell–mediated tumor suppression (24).

The formation of immune synapses between NK cells and DCs has been linked to the functional outcomes of their interactions. Polarization of IL-12 toward the NK cell–DC synapse leads to NK cell activation (25), and direct contact is required for the effective delivery of IL-18 to NK cells (16). From the DC side, polymerization of F-actin is required for the accumulation of MHC class I at the NK cell synapse and is important for survival of mature DCs (26). Studies in chemokine-deficient mice and intravital imaging point to interactions between NK cells and DCs occurring in lymph nodes during inflammation (27, 28). However, NK cell–DC cross-talk in circumstances outside Th1-promoting microbial infection or tumor responses have been studied far less.

Th2 inflammation is essential for defense against parasitic worms (helminths) (29). Infections left unchecked can cause stunted growth in children and increased maternal mortality and morbidity (30). Th2 inflammation also plays a pathogenic role in allergic asthma (31, 32), and DCs are indispensable for the development of both helminth- and allergen-induced Th2 responses (33–36). There is growing evidence that NK cells also have a part to play in the development of Th2 inflammation. In the context of helminth responses, mice infected with *Nipostrongylus brasiliensis* show expansion of B220^high^CD11b^low^ NK cells (37), and a variety of studies have implicated NK cells in the modulation of inflammation caused by *Schistosoma japonicum* (38–40). NK cells may also influence pathogenic Th2 inflammation during allergic disease, where NK cell cross-talk with DCs is disrupted (41), and an increased proportion of CD56^dim^ cells has been observed in the lungs of asthma patients (42). Despite the potential importance of both NK cells and DCs during Th2 inflammation, the effect of interactions between these cells in this context is unknown.

Thus, we developed an in vitro coculture system to compare NK cell interactions with human monocyte-derived DCs treated with Th2-polarizing stimulus *S. mansoni* soluble egg Ag (SEA) or Th1-inducing stimuli bacterial LPS or polyinosinic–polycytidylic acid [poly(I:C)]. NK cells in culture with DCs treated with SEA became activated and lysed these DCs. Blocking NK cell–activating receptors DNAM-1 and NKp30 diminished NK cell–mediated lysis of DCs treated with SEA, establishing the importance of these receptors in this process. Thus, NK cells may influence the development of Th2 inflammatory responses to schistosome eggs by lysing DCs, which polarize such responses.

## Materials and Methods

### Isolation of human primary cells

Primary human NK cells, monocytes, and naive CD4^+^ T cells were isolated from peripheral blood from healthy human donors. The blood was acquired from the National Health Service blood service under ethics licenses Research Ethics Committee 05/Q0401/108 and 2017-2551-3945 (University of Manchester). PBMCs were separated from the blood using density gradient centrifugation (Ficoll-Paque Plus; Amersham Pharmacia Biotech).

Primary human NK cells were isolated using negative magnetic bead selection (Miltenyi Biotec). After isolation, NK cells were cultured at 10^6^ cells/ml in NK cell media (DMEM with 10% human AB serum, 30% Ham F-12, 2 mM l-glutamine, 2 mM sodium pyruvate, 50 U/ml penicillin, 50 μg/ml streptomycin, 1 mM nonessential amino acids, and 20 μM 2-ME, all Sigma-Aldrich except l-glutamine and 2-ME from Life Technologies) and 200 U/ml IL-2 (Roche/PeproTech) at 37°C and 5% CO_2_. NK cells were used 6–8 d after IL-2 stimulation. T cells were isolated by negative selection using negative magnetic bead separation (Human Naive CD4^+^ T Cell Isolation Kit II; Miltenyi Biotec) and used directly for T cell coculture experiments.

CD14^+^ monocytes were isolated using human CD14 magnetic MicroBeads (Miltenyi Biotec) and cultured at 4 × 10^5^ cells/ml in RPMI 1640 medium supplemented with 10% FBS, 50 U/ml penicillin, 50 μg/ml streptomycin, 2 mM glutamine (all Sigma-Aldrich), and 25 ng/ml IL-4 and 25 ng/ml GM-CSF (BioLegend) at 37°C and 5% CO_2_ to generate monocyte-derived DCs, a method adapted from previously described protocols (43). Media were replaced after 3 d of culture, and monocyte-derived DCs were used 6–8 d after the start of culture. At this point, DCs were at least 90% CD14^−^ HLA-DR^+^. DCs were treated for 24 h with 100 ng/ml *Escherichia coli* LPS (Sigma-Aldrich), 5 μg/ml poly(I:C) (Sigma-Aldrich), 25 μg/ml *S. mansoni* SEA [generated in house as described previously (44)], or 500 ng/ml recombinant omega-1 protein [generated in *Nicotiana benthamiana* and purified from the leaf extracellular space using POROS 50 Cation Resin (Life Technologies) (45)]. For experiments with maturation factors, cells were treated as listed with the addition of 50 ng/ml recombinant human TNF-α and 20 ng/ml recombinant human IL-1β (both Miltenyi Biotec).

### Cell lines

All cells were cultured at 37°C and 5% CO_2_. 721.221 and K562 cells were maintained in RPMI 1640 medium (Sigma-Aldrich) supplemented with 10% FBS, 50 U/ml penicillin, 50 μg/ml streptomycin, and 2 mM glutamine (all from Sigma-Aldrich). All cell lines were routinely tested for mycoplasma infection using a PCR-based kit (Promocell).

### T cell polarization assay

Assays to determine T cell polarizing capability of treated DCs were adapted from published protocols (46). DCs were treated for 24 h with LPS, poly(I:C), SEA, or omega-1, then washed and plated at 3 × 10^3^ cells per well in RPMI 1640 medium (Sigma-Aldrich) supplemented with 10% FCS in a 96-well flat-bottom plate (Costar, Corning). DCs were treated with 100 ng/ml Staphylococcal enterotoxin B (Sigma-Aldrich) for 1 h, then 3 × 10^4^ allogeneic freshly isolated naive CD4^+^ T cells were added to each well. After 6 d of coculture, cells were stimulated with 10 U/ml IL-2. After 13 d, cells were restimulated with 1 μg/ml PMA and 1 μg/ml ionomycin (Sigma-Aldrich) for 6 h in the presence of brefeldin A (GolgiPlug, 1/1000 dilution; BD Biosciences) and monensin (GolgiStop, 1/1000 dilution; BD Biosciences) for the final 4 h. Cells were then stained with viability dye (Zombie NIR; BioLegend), PerCP Cy5.5–labeled anti-CD4 mAb (OKT4; BioLegend), FITC-labeled anti-CD3 mAb (UCHT1; BioLegend), and BV421-labeled anti-CD11c mAb (3.9; BioLegend), then fixed and permeabilized (Cytofix/Cytoperm Buffer; BD Biosciences) and stained with PE-labeled anti–IL-4 mAb (8D4-8; BD Biosciences) and AF647-labeled anti–IFN-γ mAb (4S.B3; BD Biosciences) or isotype-matched controls (PE IgG1k, MOPC-31C; BD Biosciences, AF647 IgG1k, MOPC-31C; BD Biosciences). Samples were assessed by flow cytometry (BD FACSCanto II Flow Cytometer; BD Biosciences) and analyzed (FlowJo V10 software). T cells were gated as live singlet CD3^+^ CD4^+^ cells before further analysis.

### DC phenotyping

DCs were washed in 1% FBS/PBS and blocked with 2% AB human serum/PBS (Sigma-Aldrich) for 20 min at 4°C before staining. Treated DCs were stained for 1 h at 4°C with viability dye (Zombie NIR; BioLegend), FITC-labeled anti-CD14 mAb (61D3; eBioscience), BV421-labeled anti-CD11c mAb (3.9; BioLegend), PerCP Cy5.5–labeled anti–HLA-DR mAb (L243; BioLegend), PE-labeled anti-CD86 (IT2.2; BioLegend), and APC-labeled anti–HLA class I mAb (W6/32; eBioscience). DCs were also stained with corresponding isotype-matched Abs (FITC-labeled mouse IgG1k P.3.6.2.8.1; eBioscience, BV421-labeled mouse IgG1k MOPC-21; BioLegend, PerCP Cy5.5–labeled mouse IgG2k MOPC-173; BioLegend, PE-labeled mouse IgG2bk MOPC-11; BioLegend, and APC-labeled mouse IgG2a eBM2a; eBioscience). To measure expression of ligands for NK cell receptors, DCs were also stained with viability dye (Zombie Aqua; BioLegend) along with either PE-labeled anti–B7-H6 mAb (875001; R&D Systems) and AF488-labeled anti-CD112 mAb (610603; R&D Systems) or PE-labeled anti-MICA/MICB mAb (6D4; Miltenyi Biotec) and AF647-labeled anti-CD155 mAb (SKII.4; BioLegend). DCs were also stained with corresponding isotype-matched Abs (PE mouse IgG1k 11711; R&D, AF488 mouse IgG1k 11711; R&D, PE mouse IgG2a S43.10; Miltenyi Biotec, and AF647 mouse IgG1k MOPC21; BioLegend). Samples were assessed by flow cytometry (BD FACSCanto II Flow Cytometer; BD Biosciences) and analyzed (FlowJo V10 software). DCs were gated as live singlet CD14^–^CD11c^+^ cells before further analysis.

### Degranulation and apoptosis assays

For degranulation assays, NK cells and treated DCs were coincubated at a 1:1 ratio in the presence monensin (GolgiStop, 1/1000 dilution; BD Biosciences) and brefeldin A (GolgiPlug, 1/1000 dilution; BD Biosciences) and an AF647-labeled anti–LAMP-1 mAb (H4A3; Santa Cruz Biotechnology) or isotype-matched control (AF647 IgG1k MOPC-21; BioLegend) for 5 h at 37°C in RPMI 1640 media (Sigma-Aldrich) with 10% FBS. Cells were washed with PBS with 2% FBS and 2 mM EDTA to separate conjugates and were stained with viability dye (Zombie NIR; BioLegend), AF647-labeled anti–LAMP-1 mAb (H4A3; Santa Cruz Biotechnology) or isotype-matched control (AF647 IgG1k MOPC-21; BioLegend), BV421-labeled anti-CD11c mAb (3.9; BioLegend), and PE-labeled anti-CD56 mAb (HCD56; BioLegend). Stained cells were assessed by flow cytometry (BD FACSCanto II Flow Cytometer; BD Biosciences).

To measure apoptosis of DCs in cocultures, treated DCs were washed and labeled with cell tracing dye (CellTrace Violet; Thermo Fisher), then cultured with NK cells at a 1:1 ratio for 5 h. Cells were washed with PBS and stained at room temperature for 20 min with FITC-labeled annexin V (BD Biosciences) and TO-PRO-3 Iodide (Thermo Fisher) in dH_2_O with 2.5 mM CaCl_2_, 140 mM NaCl, and 10 mM HEPES. Stained cells were assessed by flow cytometry (BD FACSCanto II Flow Cytometer; BD Biosciences). Where indicated, hybridoma supernatants containing IgM blocking Abs to NKp30 (F252) and DNAM-1 (F5) or isotype-matched control (IgM, G155-228; BD Biosciences) were added to NK cells 1 h before the start of cocultures and included in coculture media.

### ^35^S release killing assays

Lysis of DCs and target cell lines was measured by standard [^35^S]methionine release assays, as described previously (47). Before coculture, 5 × 10^5^ cells treated DCs and K562 cells were cultured overnight in 750 μl of methionine-free RPMI 1640 medium with 10% FBS (Sigma-Aldrich) with 0.37 MBq of radiolabeled ^35^S (Perkin Elmer). NK cells were added to targets at E:T ratios of 10:1, 5:1, and 1:1. Release of ^35^S was measured in counts per minute after overnight incubation of 5-h culture supernatants with scintillation fluid (MicroScint 40; Perkin Elmer) using a scintillation counter (MicroBeta2 Microplate Counter; Perkin Elmer). Spontaneous lysis of DCs cultured alone and total lysis of DCs treated with 10% TritonX-100 was also measured. Specific lysis was calculated as follows:

% lysis=(cpm experimental well −– cpm spontaneous)/(cpm total –− cpm spontaneous)

Only experiments in which the spontaneous/total lysis ratio was below 25% were analyzed.

Where indicated, hybridoma supernatants containing IgM blocking Abs to NKp30 (F252) and DNAM-1 (F5) or IgM isotype control (G155-228; BD Biosciences) were added to NK cells 1 h before the start of cocultures and included in coculture media.

### DC imaging

Preparation of eight-chamber glass coverslips was adapted from previous protocols (48). Briefly, slides (no. 1.5 Lab-Tek II; Nunc) were coated with 0.01% poly-l-lysine, dried, and coated with 10 μg/ml fibronectin (Sigma-Aldrich) for 1 h at room temperature. A total of 10^4^ immature DCs or DCs treated for 24 h with LPS or SEA were plated in each chamber and allowed to spread for 1 h at 37°C and 5% CO_2_. DCs were fixed with 4% PFA/PBS and then blocked for 1 h at room temperature with PBS/2% human serum (Sigma-Aldrich). DCs were stained for F-actin with AF488-labeled phalloidin dye (Thermo Fisher). Samples were imaged by stimulated emission depletion microscopy with a 100×/1.40 oil immersion objective (Leica TCS SP8). Images were exported to ImageJ (National Institutes of Health) for analysis. For interference reflection microscopy and cell migration analysis, DCs were plated on fibronectin-coated coverslips and imaged live at 37°C and 5% CO_2_ over 15 min–5 h with either a 100×/1.40 oil immersion objective (for 15 min images) or a 20×/0.75 air immersion objective (for 5-h cell tracking). DC migration was tracked from brightfield images using open-source software [CellTracker and MSD analyzer (49, 50)].

### NK cell–DC conjugate imaging

Glass coverslips (1.5, 18 mm; VWR) were cleaned by sonicating for 15 min in acetone and 15 min in 1 M NaOH, then rinsed with dH_2_O and coated with 0.01% poly-l-lysine. NK cells and treated DCs were suspended at 1 × 10^7^ cells/ml, 10 μl of each combined at a 1:1 ratio and spun down at 200 g for 1 min, then incubated together for 5 min at 37°C and fixed with 4% PFA/PBS for 20 min at 37°C. Conjugates were permeabilized with 0.1% Triton X-100 for 5 min and then blocked for 1 h at room temperature in PBS/3% BSA/2% human serum (all Sigma-Aldrich). Fixed conjugates were stained either with AF647-labeled anti–LFA-1 mAb (HI111; eBioscience), then AF488-labeled phalloidin (1/200 dilution in PBS; Invitrogen) for LFA-1 imaging. For microtubule organizing center (MTOC) imaging, conjugates were stained with antipericentrin polyclonal Ab (ab4448; Abcam) and anti-NKp30 mAb (P30-15; BioLegend) followed by secondary Abs AF594-labeled goat anti-rabbit IgG H&L (ab150084; Abcam) and goat anti-mouse IgG H&L (ab21121; Invitrogen), then AF647-labeled phalloidin (1/200 dilution in PBS; Invitrogen). All Abs were incubated for 1 h at room temperature, and phalloidin (marking F-actin) was incubated at room temperature for 20 min. Stained conjugates were plated on PLL-coated coverslips and attached to glass slides (clarity 1 mm C362) with low viscosity mounting medium (Dako, Agilent).

Samples were imaged by confocal microscopy (Leica TCS SP8) with a 100×/1.40 oil immersion objective. Images were exported to ImageJ (National Institutes of Health) for analysis. Polarization of LFA-1 to the synapse was determined by the fold increase in mean fluorescence intensity (MFI) of staining at the intercellular contact divided by the MFI at the back of the NK cell. Polarization of the MTOC was determined by measuring the distance from the MTOC to the cell interface and dividing this by the distance from the intercellular contact to the back of the cell.

### Time-lapse microwell imaging of NK cell–DC interactions

Cells were imaged by time-lapse microscopy in custom-made silicone glass microchips with wells of dimensions 450 × 450 × 300 μm using methods adapted from previously published protocols (51). Briefly, sterile microchips were blocked with filtered RPMI 1640 media supplemented with 10% FBS (Sigma-Aldrich). Primary NK cells were stained with 1 μM calcein red-orange, and treated DCs were labeled with both 0.3 μM calcein green and 0.64 μM CellTrace Far Red (all Thermo Fisher) for 20 min at 37°C. DCs were first added to wells to obtain an even distribution of cells, then NK cells were added to the wells in filtered RPMI 1640 with 10% FBS (Sigma-Aldrich) containing 1 μM SYTOX Blue Dead Cell Stain (Thermo Fisher). Images were obtained with 20×/0.80 air immersion objective using an inverted confocal microscope equipped with a motorized stage (LSM 880; Zeiss). By using polydimethylsiloxane gaskets, the microchip was divided into four separate areas, each consisting of multiple wells, making it possible to record time-lapse sequences from multiple positions and conditions in a single experiment. Interactions of NK cells with DCs treated for 24 h with LPS, poly(I:C), or SEA were imaged at 37°C and 5% CO_2_ for 4–6 h, with one image per well acquired every 3 min. For experiments comparing NK cell responses to DCs marked by how much Ag they have taken up, DCs were treated for 24 h with 25 μg/ml AF488-labeled SEA (Thermo Fisher) and then labeled with a cell tracing dye (CellTrace Far Red; Thermo Fisher). SEA was labeled by incubating the extract with AF488 NHS Ester, then removing free dye using Sephadex columns (Nap-5; GE Healthcare). Images were analyzed using ImageJ (National Institutes of Health). NK cell contacts were defined as a flattened interface between an NK cell and a DC lasting longer than 1 frame (3 min).

### Statistics

For each data set, a D’Agostino–Pearson omnibus test was used to evaluate the distribution of values. The statistical significance of differences between groups of data with normal Gaussian distribution was determined by one-way ANOVA or two-way ANOVA. For matched values, repeated measures one-way ANOVA with Tukey multiple comparisons or repeated measures two-way ANOVA with Sidak/Dunnett multiple comparisons were used. If at least one of the conditions did not have a normal distribution, nonparametric tests were used. Multiple comparisons were evaluated using a Kruskal–Wallis test or matched values Friedman test with Dunn posttesting. Differences were defined as not significant where *p* ≥ 0.05 and statistically significant where *p* < 0.05 [indicated by a single asterisk (*)], **p < 0.01, ***p < 0.001, and ****p < 0.0001. For values quoted in the text, SD of the mean is given unless stated otherwise. All statistical analysis was performed using Prism (version 7; GraphPad Software).

## Results

### DCs polarize Th2 responses after treatment with S. mansoni *soluble egg Ag*

**FIGURE 1.**
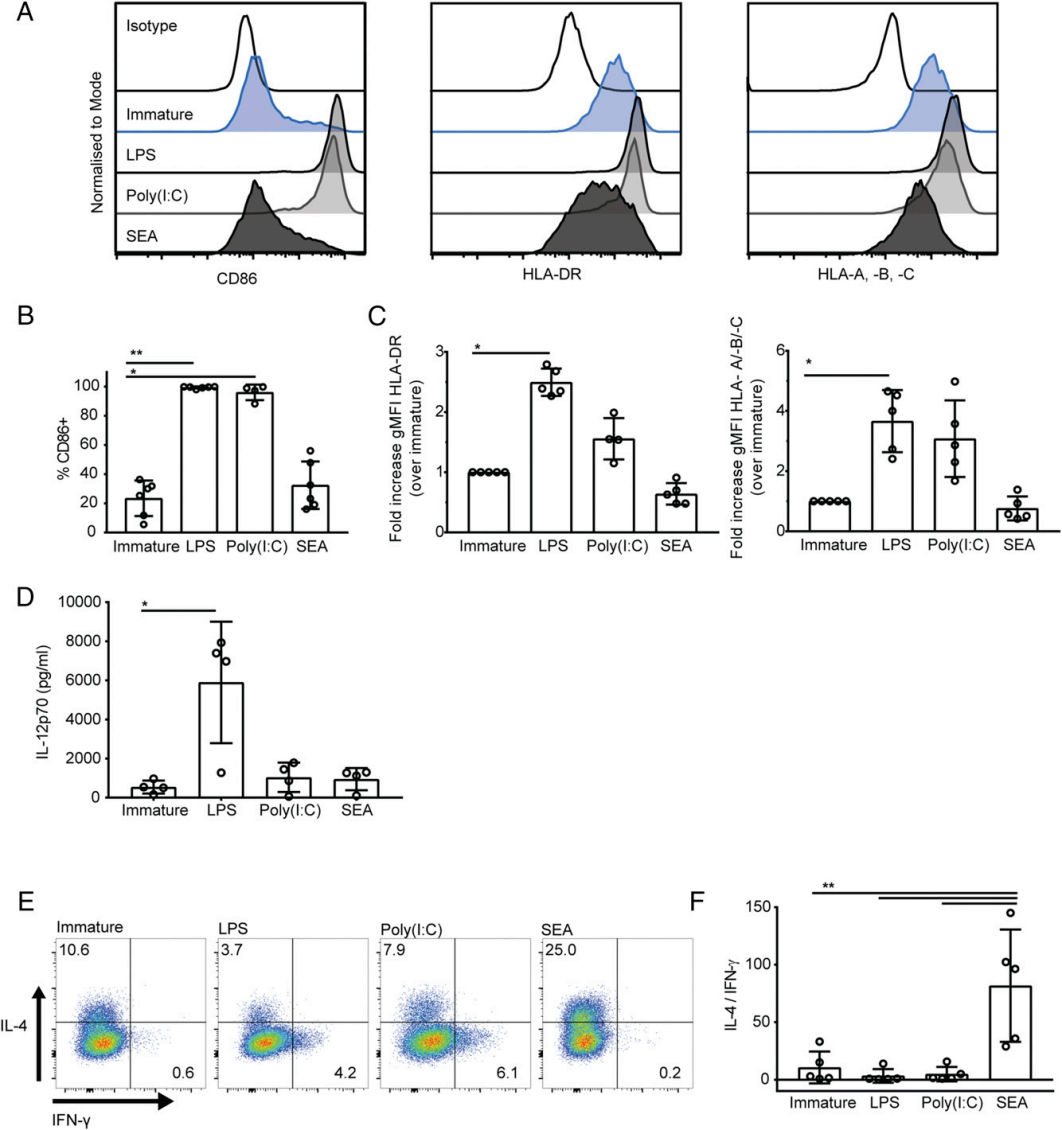
DCs treated with SEA polarize Th2 responses in vitro. (**A**) Expression of CD86, HLA-DR, and HLA-A, -B, and -C on the surface of immature DCs (blue, second row) and DCs treated for 24 h with LPS (gray, third row), poly(I:C) (gray, fourth row), or SEA (black, bottom row) compared with isotype-matched control staining (top row). Data from one representative donor of six tested. (**B**) Percentage of DCs expressing CD86, comparing immature DCs and DCs treated for 24 h with LPS, poly(I:C), or SEA. (**C**) Fold change in gMFI of HLA-DR (left) and HLA-A, -B, and -C (right) in DCs after 24-h treatment with LPS, poly(I:C), or SEA, normalized to the level of expression in immature DCs. (**D**) Concentration of IL-12p70 in supernatants of immature DCs and DCs treated for 24 h with LPS, poly(I:C), or SEA. (**E**) IL-4 and IFN-γ production by CD4^+^ T cells stimulated with PMA and ionomycin after 13-d culture with treated DCs in the presence of staphylococcal enterotoxin B; dot plots from one representative donor (of five tested) gated on live singlet CD4^+^ T cells. (**F**) Ratio of IL-4–positive over IFN-γ–positive CD4^+^ T cells. In all plots, circles represent data points from individual donors and bars show mean (±SD) of four to six independent donors. *p < 0.05, ** *p* < 0.01, analyzed by Kruskal–Wallis test with Dunn multiple comparisons (B–D) and repeated measures one-way ANOVA with Tukey multiple comparisons (E).

DCs stimulated with microbial pathogen-associated molecular patterns show changes in surface receptor expression and induce Th1- and Th2-polarized responses from naive CD4^+^ T cells (52). To generate DCs capable of polarizing a Th2 response in vitro, we treated human monocyte-derived DCs with helminth *S. mansoni* SEA for 24 h. To generate DCs capable of polarizing a Th1 response, we treated DCs with either bacterial LPS or poly(I:C). To assess the DC maturation state after each type of stimulation, immature DCs or DCs treated for 24 h with LPS, poly(I:C), or SEA were stained with Abs to the costimulatory molecule CD86 as well as MHC class I and class II molecules (HLA-A, -B, -C and HLA-DR) and assessed by flow cytometry. After 24-h treatment, 99 ± 1% of DC treated with LPS and 93 ± 8% of DCs treated with poly(I:C) stained positive for CD86, compared with 23 ± 12% of immature DCs and 32 ± 16% of DCs treated with SEA (Fig. 1A, 1B). DCs treated with LPS also showed a significant increase in the geometric MFI (gMFI) of staining for HLA-DR and HLA-A, -B, and -C (Fig. 1A, 1C). In contrast, immature DCs and DCs treated for 24 h with SEA did not show an increase in staining for MHC class I or II proteins (Fig. 1A, 1C). Indeed, a small decrease in expression of these proteins is apparent in SEA-treated DCs, although not to a statistically significant extent. Thus, DCs treated with LPS and poly(I:C) show increased expression of CD86 and MHC receptors, whereas DCs treated with SEA did not significantly increase expression of these markers.

The proinflammatory cytokine IL-12 is particularly important in the polarization of Th1 responses, therefore we tested the ability of LPS, poly(I:C), or SEA to induce DC secretion of the biologically active form of the cytokine, IL-12p70. Supernatants from immature DCs and DCs treated for 24 h with LPS, poly(I:C), or SEA were tested for IL-12p70 by ELISA. DCs secreted significant levels of IL-12p70 (Fig. 1D), whereas neither SEA nor poly(I:C) provoked IL-12p70 secretion above amounts detected in supernatants from immature DCs, as has been observed previously (52, 53).

To test whether these differently treated DCs could polarize Th1 or Th2 responses in vitro, DCs were coincubated with naive CD4^+^ T cells in the presence of stimulating superantigen staphylococcal enterotoxin B (46). After 13-d culture, T cell production of Th1 cytokine IFN-γ and Th2 cytokine IL-4 was assessed by intracellular staining and flow cytometry. To account for donor variability in the absolute levels of cytokine produced, the polarization of the T cell response was measured as a ratio of the number of T cells producing IL-4 versus IFN-γ. T cells cultured with DCs treated with SEA showed a significant increase in the ratio of cells producing IL-4/IFN-γ compared with T cells cultured with either immature DCs or DCs treated with LPS or poly(I:C) (Fig. 1E, 1F). This confirms that DCs treated for 24 h with SEA preferentially induce a Th2 response in vitro, despite not upregulating MHC class II and costimulatory molecules commonly used to describe phenotypic maturation of DCs.

**FIGURE 2.**
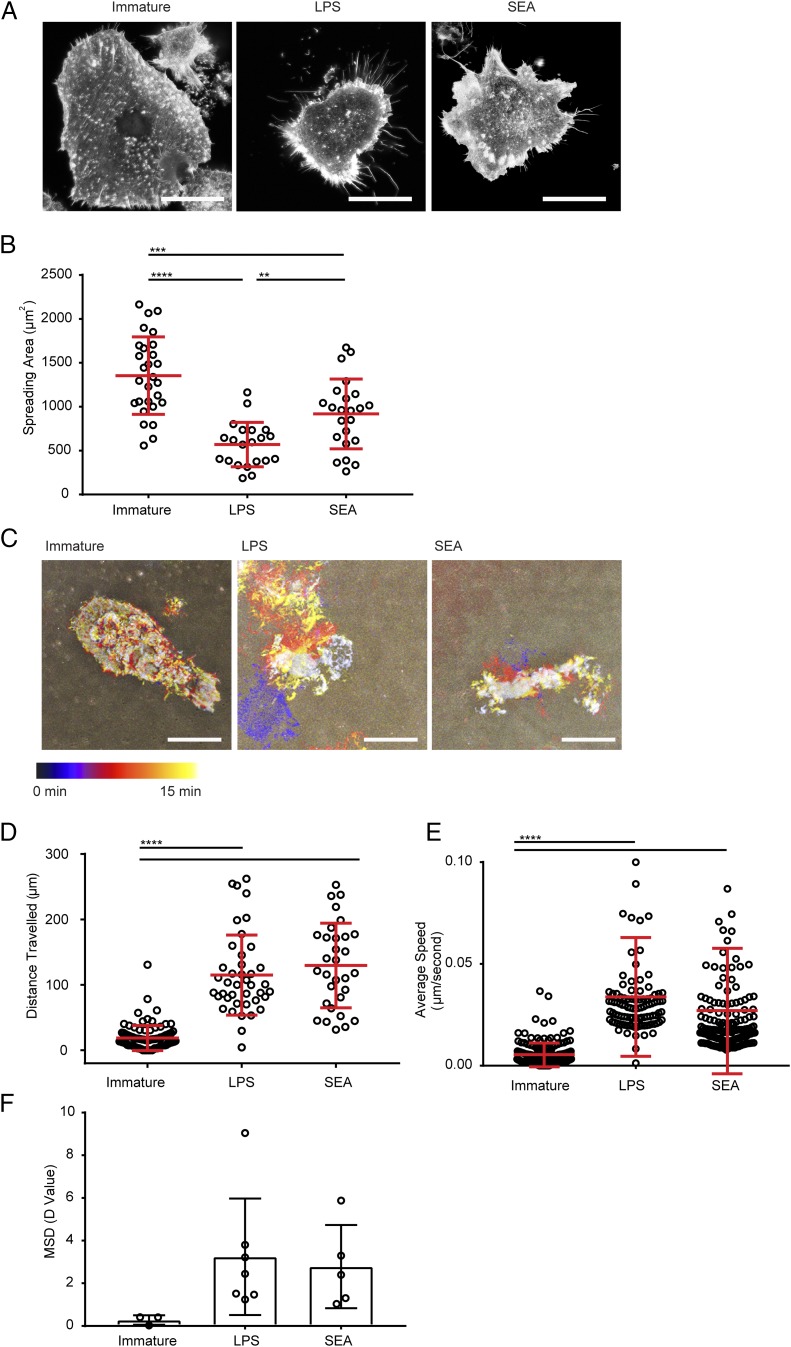
DCs treated with SEA show distinct morphology and spreading behavior. (**A**) Fluorescent images of immature DCs or DCs treated for 24 h with LPS or SEA, incubated on 10 μg/ml fibronectin, and stained with AF488-labeled phalloidin (to mark F-actin). Representative from 50 images taken with three independent donors; scale bar, 20 μm. (**B**) Spreading area of DCs measured from images as in (A) pooled from three donors. (**C**) Time-lapse interference reflection microscopy (IRM) images of DCs spreading on 10 μg/ml fibronectin over 15 min. Images show differently silhouettes of the adherent membrane color coded by time. Data are representative of 8–10 images obtained with three independent donors. (**D**) Total distance traveled by immature DCs or DCs treated for 24 h with LPS or SEA, plated on 10 μg/ml fibronectin, and tracked by time-lapse imaging over 5 h. (**E**) Average migration speed of immature DCs or DCs treated for 24 h with LPS or SEA, plated on 10 μg/ml fibronectin, and tracked by time-lapse imaging over 5 h. (**F**) Mean squared displacement (MSD) diffusion coefficient values calculated from multiple tracks of immature DCs or DCs treated for 24 h with LPS or SEA and plated on 10 μg/ml fibronectin for 5 h. In (B), (D), and (E), circles represent data points from individual cells; lines show mean (±SD) of cells pooled from three independent donors. In (F), circles represent MSD of imaging regions from three independent experiments; bars show mean (±SD) analyzed by one-way ANOVA with Tukey multiple comparisons (no significant differences). **p < 0.01, ***p < 0.001, ****p < 0.0001, analyzed by one-way ANOVA with Tukey multiple comparisons (B) and Kruskal–Wallis test with Dunn multiple comparisons (D and E).

To test for changes in the behavior of differently treated DCs, we next used fluorescence and interference reflection microscopy to compare their morphology and migration. For this, DCs were plated on fibronectin-coated surfaces for 1 h, then fixed and labeled with a stain for F-actin. DCs treated with SEA showed a reduced spreading area compared with immature DCs, as did DCs treated with LPS (Fig. 2A, 2B). DCs were also imaged live by time-lapse microscopy to track their movement on fibronectin-coated surfaces. DCs treated with SEA traveled farther and faster than immature DCs, as did DCs treated with LPS (Fig. 2C–F). These results show that cell morphology and movement distinguish Th1- and Th2-polarizing DCs from immature DCs.

### NK cells form immune synapses with treated DCs

**FIGURE 3.**
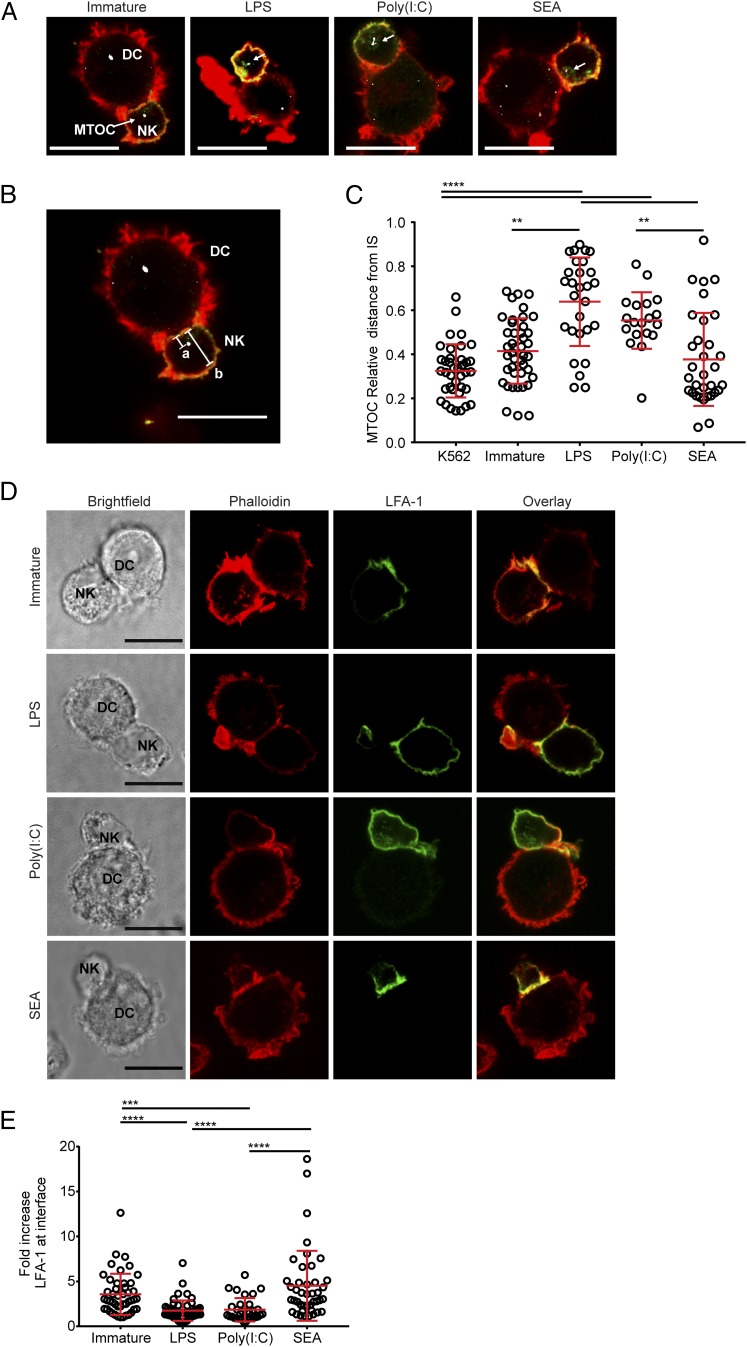
NK cells show MTOC and LFA-1 reorganization in conjugates with DCs treated with SEA. (**A**) Panels show autologous NK cells conjugated with immature DCs (left) or DCs treated for 24 h with LPS, poly(I:C), or SEA, overlays of cells stained with phalloidin (red), anti-NKp30 mAb (green), and antipericentrin polyclonal Ab (white). Representative data from >150 images taken over three independent experiments; scale bar, 10 μm. (**B**) Example of MTOC distance measurement with image from (A). Distance from MTOC to cell–cell interface (line labeled as a) is divided by the diameter of the NK cell (line labeled as b). (**C**) Relative distance of the NK cell MTOC from immune synapse in conjugates with K562 target cells, immature DCs, or DCs treated for 24 h with LPS, poly(I:C), or SEA. (**D**) Panels show images of autologous NK cells conjugated with immature DCs or DCs treated for 24 h with LPS, poly(I:C), or SEA stained with phalloidin (second column, red) and anti–LFA-1 mAb (third column, green). A brightfield image (left) and overlay of the two channels (right, yellow shows colocalization) are also shown. Images representative of >150 images taken across three independent experiments; scale bar, 10 μm. (**E**) Fold increase in fluorescence intensity of staining with anti–LFA-1 mAb at the cell interface, compared with the back of the NK cell, in conjugates with immature DCs and DCs treated for 24 h with LPS, poly(I:C), or SEA. In all plots, circles represent data points from individual cell contacts pooled from three independent donors; lines show mean (±SD). **p < 0.01, ***p < 0.001, ****p < 0.0001, analyzed by Kruskal–Wallis test with Dunn multiple comparisons.

Having established that DCs treated with SEA are able to polarize Th2 responses and show distinct adherence and movement from immature DCs, we set out to probe their interactions with NK cells. Immature DCs or DCs treated for 24 h with LPS, poly(I:C), or SEA were conjugated with autologous NK cells, stained with phalloidin (to label F-actin) and Abs to NKp30 and pericentrin (to mark the MTOC), and imaged by confocal microscopy (Fig. 3A). For comparison, NK cells were also incubated with K562, a human leukemic cell line well established to be efficiently lysed by NK cells (54). The NK cell MTOC clearly polarized to the immune synapse with K562 targets as expected (55–57). In addition, the NK cell MTOC polarized to the synapse with immature DCs or Th2-polarizing DCs but not with Th1-polarizing DCs (Fig. 3A–C). The MTOC did not move toward the synapse in conjugates with DCs treated with LPS or poly(I:C), as has been previously demonstrated for these mature DCs (26).

NK cell–DC conjugates were also stained with Abs to the CD11a component of integrin LFA-1, which is reorganized to the interface between cells in NK cell lytic synapses (58). NK cells in conjugates with immature DCs or DCs treated with SEA, but not DCs treated with LPS or poly(I:C), showed an increased accumulation of LFA-1 staining at the interface (Fig. 3D, 3E). These results, taken together with the observation that the NK cell MTOC moved toward the synapse with DCs treated with SEA, indicate the assembly of an activating NK cell synapse with immature or Th2-polarizing DCs.

### NK cells lyse immature and Th2-polarizing DCs

**FIGURE 4.**
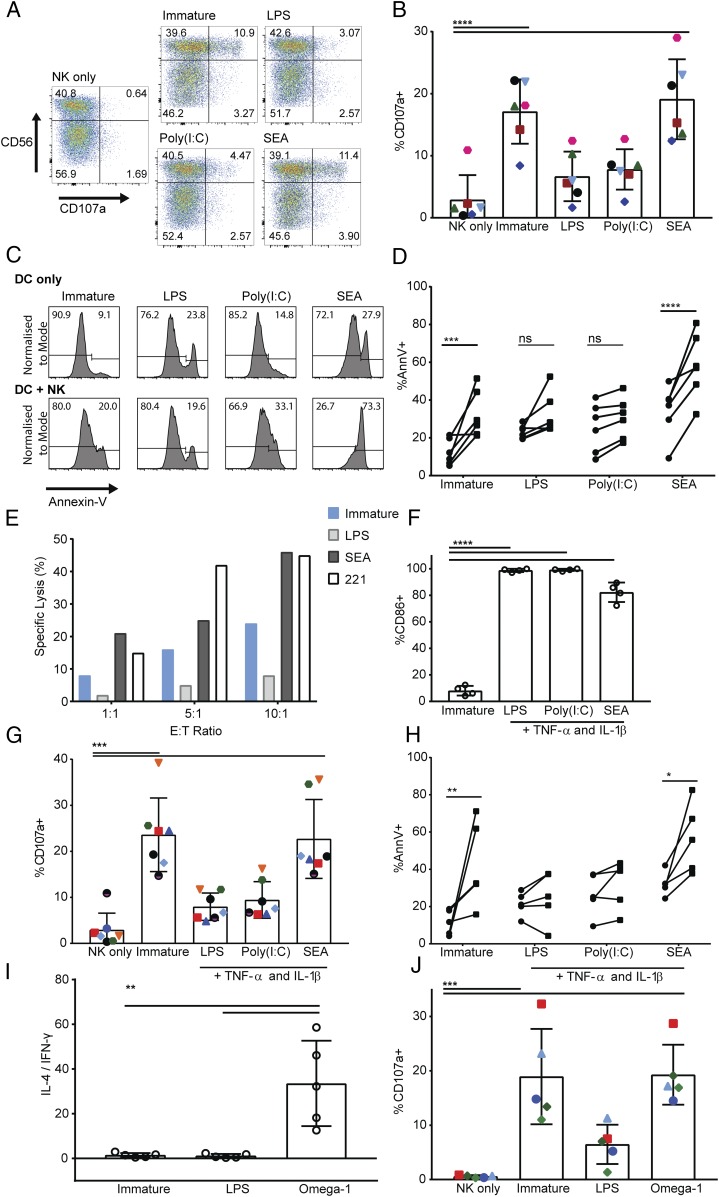
NK cells induce apoptosis of DCs treated with SEA. (**A**) NK cell expression of CD107a after 5-h culture alone (NK only) or at a 1:1 ratio with immature DCs or DCs treated for 24 h with LPS, poly(I:C), or SEA. Plots show analysis of CD107a and CD56 gated on live singlet NK cells; representative from one donor of six tested. (**B**) Total percentage of CD107a-positive NK cells after 5-h culture alone (NK only) with immature DCs or DCs treated with LPS, poly(I:C), or SEA; points of the same shape/color show measurements from the same donor. Bars show mean (±SD) of data pooled from six donors. (**C**) Annexin V staining of DCs cultured alone (top) or with autologous NK cells at a 1:1 ratio (bottom) for 5 h. Histograms from one representative donor showing the proportion of annexin V–negative (left) and –positive populations (right) of DCs. (**D**) Difference in annexin V staining of DCs cultured alone (circles) compared with DCs cultured with autologous NK cells at a 1:1 ratio for 5 h (squares). Connected data points show paired measurements from six independent donors. (**E**) Specific lysis of 221 target cells, immature DCs, or DCs treated for 24 h with LPS or SEA by autologous NK cells at 1:1, 5:1, and 10:1 NK:DC (E:T) ratios measured by release of ^35^S over 5 h. Plots show data from one representative donor of three; mean of values measured in triplicate. (**F**) Percent of DCs expressing CD86 after treatment for 24 h with LPS, poly(I:C), or SEA in the presence of 50 ng/ml TNF-α and 20 ng/ml IL-1β as maturation factors. (**G**) The proportion of NK cells stained positive for CD107a after 5-h culture with immature DCs or DCs that had previously been treated for 24 h with LPS, poly(I:C), or SEA in the presence of maturation factors TNF-α and IL-1β. (**H**) The difference in annexin V staining of immature DCs or DCs treated with LPS, poly(I:C), or SEA in the presence of maturation factors TNF-α and IL-1β cultured alone (circles) compared with DCs cultured with autologous NK cells at a 1:1 ratio for 5 h (squares). Connected data points show paired measurements from five independent donors. (**I**) IL-4 and IFN-γ production by CD4^+^ T cells stimulated with PMA and ionomycin after 13-d culture with immature DCs, LPS, or omega-1–treated DCs in the presence of staphylococcal enterotoxin B; ratio of IL-4–positive over IFN-γ–positive CD4^+^ T cells. (**J**) Total percentage of CD107a-positive NK cells after 5-h culture alone (NK only) with immature DCs or DCs previously treated with LPS or omega-1; points of the same shape/color show measurements from the same donor. Bars show mean (±SD) of data pooled from five donors. In all plots, circles represent data points from individual donors, and bars show mean (±SD) of three to six independent donors. *p < 0.05, **p < 0.01, ***p < 0.001, ****p < 0.0001, analyzed by repeated measures one-way ANOVA with Tukey multiple comparisons (B, G, and J), one-way ANOVA with Dunnett multiple comparisons (F), and repeated measures two-way ANOVA with Sidak multiple comparisons (D and H).

We next tested the functional outcome of NK cell interactions with differently treated DCs. First, immature DCs or DCs treated with LPS, poly(I:C), or SEA were cultured with autologous NK cells, after which NK cells were stained for surface expression of CD107a, an indicator of degranulation (59). Only 3 ± 4% of NK cells cultured alone stained positive for CD107a. In contrast, NK cells cultured with immature DCs or DCs treated with SEA showed a significant increase in the percentage of NK cells stained positive for CD107a, rising to 16 ± 6% and 18 ± 7%, respectively (Fig. 4A, 4B). This was not the case for NK cells cultured with DCs treated with LPS or poly(I:C), which showed no significant increase in the proportion of NK cells stained positive for CD107a compared with NK cells cultured alone (Fig. 4A, 4B). These data indicate that NK cell degranulation marked by CD107a is triggered by immature DCs and Th2-polarizing DCs but not Th1-polarizing DCs.

To assess whether the observed NK cell degranulation resulted in lysis of DCs, we stained DCs in culture with NK cells for levels of apoptosis. DCs were treated with LPS, poly(I:C), or SEA, then labeled with a cell tracking dye, cultured alone or with autologous NK cells, and then stained for annexin V, a marker of apoptosis (Fig. 4C). Over six donors, a mean of 32 ± 12% immature DCs were stained with annexin V after 5-h culture with autologous NK cells compared with 12 ± 7% immature DCs cultured alone (Fig. 4D). For DCs treated with SEA, LPS, or poly(I:C) or cultured alone, there was no significant difference in their viability or their expression of stress-induced NK cell–activating ligands MICA/B (Supplemental Fig. 1). However, although the addition of NK cells to cultures did not significantly increase the proportion of annexin V–stained DCs that had previously been treated with LPS or poly(I:C), the proportion of annexin V–stained DCs treated with SEA increased from 34 ± 13% when cultured alone to 58 ± 17% when cultured with NK cells (Fig. 4D). These data show that immature DCs or DCs treated with SEA display increased apoptosis in culture with NK cells compared with DCs treated with LPS or poly(I:C).

To directly assess the extent that differently treated DCs are lysed by NK cells, we next performed standard ^35^S radioactive release assays. Immature DCs or DCs treated with LPS, poly(I:C), or SEA were cultured in media containing ^35^S-labeled methionine overnight, then cultured with autologous NK cells. For comparison, 721.221, an EBV-transformed cell line selected for deficiency in class I MHC expression, was also included as a target. Specific lysis of target cells was calculated from the counts per minute of ^35^S in the media. At all E:T ratios tested, there was specific lysis of control 721.221 target cells, immature DCs, or DCs treated with SEA (Fig. 4E).

To test whether NK cell–mediated lysis of DCs treated with SEA related to their lack of expression of conventional maturation markers for DCs, these experiments were repeated with DCs that had been treated with LPS, poly(I:C), or SEA in the presence of maturation factors TNF-α and IL-1β. Including these factors increased the expression of CD86 on the surface of DCs treated with SEA (Fig. 4F). However, NK cells still degranulated in cultures with DCs treated with SEA plus TNF-α and IL-1β, evidenced by their increased surface expression of CD107a (Fig. 4G). Culture of autologous NK cells with DCs treated with SEA and maturation factors also resulted in a significant increase in the proportion of apoptotic DCs marked by annexin V staining (Fig. 4H).

Because the components of SEA that polarize Th2 responses are not fully characterized, we extended these experiments to examine DCs that had been treated with recombinant omega-1. Omega-1 is a glycoprotein present in both SEA and excretory/secretory products from live *S. mansoni* eggs, which also initiates strong Th2 responses (45, 60). DCs treated with omega-1 induced naive CD4^+^ T cells to produce IL-4 and suppress IFN-γ production, indicative of Th2 polarization (Fig. 4I). DCs treated with omega-1 induced a significant increase in the proportion of autologous NK cells expressing CD107a at their surface, a marker of degranulation (Fig. 4J). Together, these data indicate that autologous NK cells are activated by immature DCs and schistosome egg–activated Th2-polarizing DCs but not Th1-polarizing DCs.

### SEA uptake correlates with NK cell lysis of DCs

**FIGURE 5.**
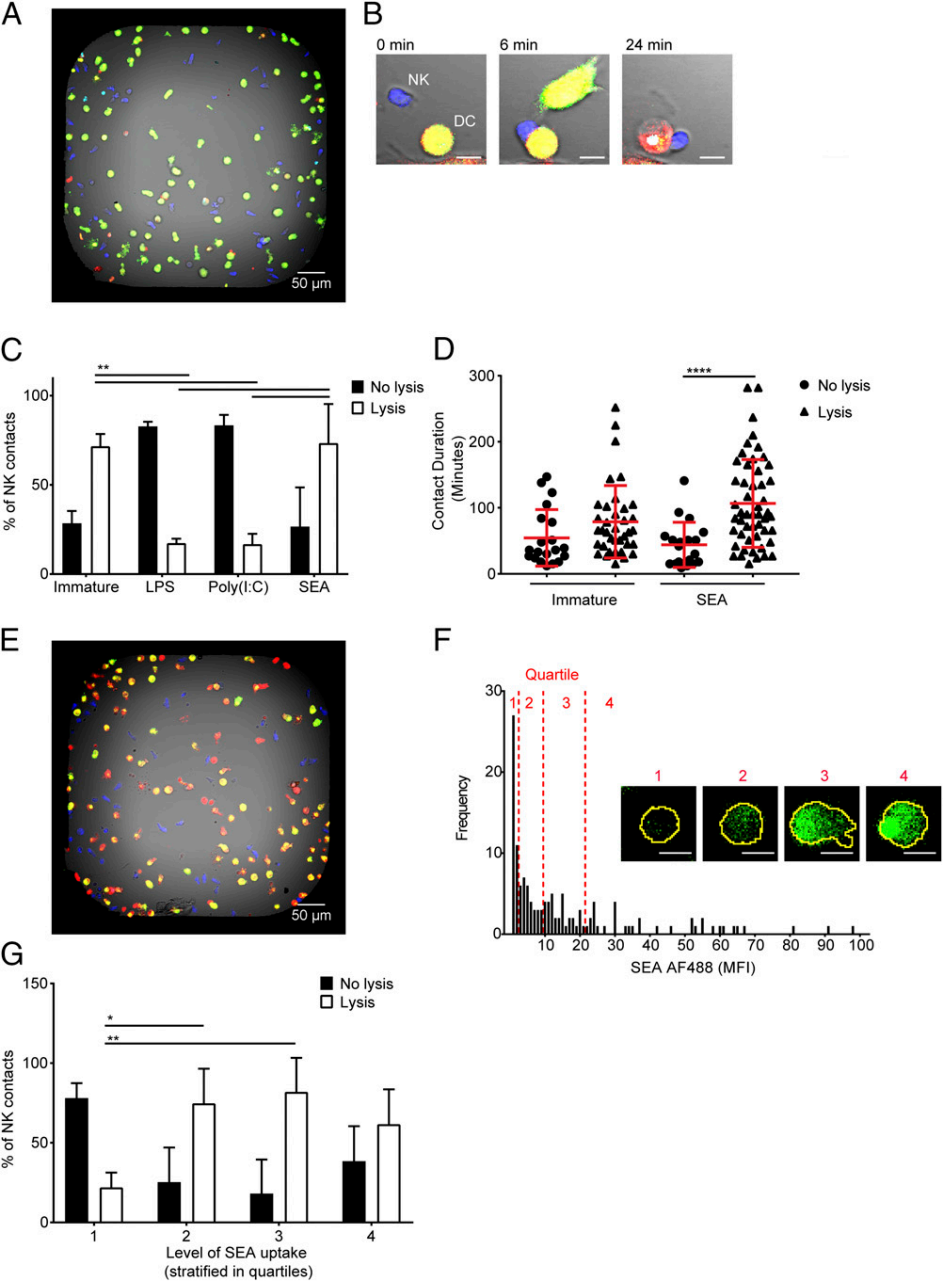
DCs that take up SEA are preferentially lysed by NK cells. (**A**) Representative image of dye-labeled DCs (green) and NK cells (blue) coincubated in a bespoke microwell chip; scale bar, 50 μm. (**B**) An example time-lapse sequence showing an NK cell (blue) forming a contact with a DC (green). Upon lysis, the DC turns from green/yellow to red (24 min); scale bar, 10 μm. (**C**) Proportion of NK cell contacts with immature DCs or DCs treated for 24 h with LPS, poly(I:C), or SEA resulting in DC survival (no lysis, filled bars) or death (lysis, empty bars). (**D**) Duration of contacts formed between NK cells and immature DCs and DCs treated for 24 h with SEA resulting in either DC survival (no lysis, circles) or DC death (lysis, triangles). (**E**) Dye-labeled DCs (red) and NK cells (blue) cocultured in a microwell after DCs were pretreated for 24 h with AF488-labeled SEA (green). (**F**) Distribution of MFI of SEA AF488 inside DCs from one representative well of nine imaged over three independent experiments. Quartiles in the level of SEA staining are marked in red with representative images of DCs labeled with AF488 SEA (green) from the first (1), second (2), third (3), or fourth (4) quartiles; scale bar, 10 μm. (**G**) Proportion of NK cell contacts resulting in survival (no lysis, filled bars) or death (lysis, empty bars) of DCs in the first (1), second (2), third (3), or fourth (4) quartiles in amount of AF488-labeled SEA they have taken up. In (C) and (G), bars show mean (±SD) from three independent donors with at least 40 cells per condition per donor, analyzed by two-way ANOVA with Tukey multiple comparisons. In (D), shapes represent individual NK cell–DC contacts with lines showing mean (±SD) of data pooled from three independent donors, analyzed by Kruskal–Wallis test with Dunn multiple comparisons. *p < 0.05, **p < 0.01, ****p < 0.0001.

We next set out to examine the dynamics of NK cell interactions with DCs by live imaging of individual NK cell–DC contacts, using a methodology published previously (51). Immature DCs or DCs treated with LPS, poly(I:C), or SEA were labeled with one fluorescent dye (CellTrace Far Red) and then stained with a second fluorescent dye (calcein green), which fluoresces brightly only in live cells. NK cells stained with a different fluorescent dye (calcein red-orange) were separately coincubated with each type of DC in the presence of a cell death dye (SYTOX Blue) in custom-made microwell chips (wells of 450 × 450 × 300 μm) prior to imaging (Fig. 5A). After formation of a contact between the NK cell (blue) and the DC (green), lysis was determined by a color change in DCs from green/yellow to red and through the accumulation of a dead cell dye (example shown in Fig. 5B). Each NK cell contact was assessed as to whether it led to lysis of the DC. Contacts with immature DCs resulted in DC death in 71 ± 7% of cases (Fig. 5C). In contrast, only 17 ± 3% of NK contacts with DCs treated with LPS and 17 ± 6% of NK contacts with DCs treated with poly(I:C) lead to lysis of the DC. Most importantly, DCs treated with SEA were lysed in 73 ± 22% of NK cell contacts (Fig. 5C). The duration of NK cell contacts with DCs treated with SEA increased when the DC was lysed, but there was no significant difference in the duration of lytic contacts between NK cells and immature DCs or DCs treated with SEA (Fig. 5D).

It was possible that the activation of NK cells in response to DCs treated with SEA was the result of NK cells responding to immature DCs left within the cultures that had not taken up SEA. To test this, we treated DCs with fluorescently labeled SEA before adding them to the microwells. DCs were divided into quartiles based on the extent of Ag uptake as measured by the cell’s fluorescence intensity (Fig. 5E, 5F). NK cell interactions with DCs in the first quartile, representing low or no Ag uptake, resulted in lysis in only 22 ± 9% of contacts. A significantly greater proportion of NK cell contacts resulted in lysis of DCs in the second and third quartiles, 74 ± 21% and 82 ± 21%, respectively (Fig. 5G). These data establish that NK cells do specifically respond to DCs that have taken up SEA. Thus, taken together, these results demonstrate that autologous NK cells kill both immature DCs and schistosome egg–stimulated Th2-polarizing DCs but not Th1-polarizing DCs.

### NKp30 and DNAM-1 contribute to NK cell lysis of Th2-polarizing DCs

**FIGURE 6.**
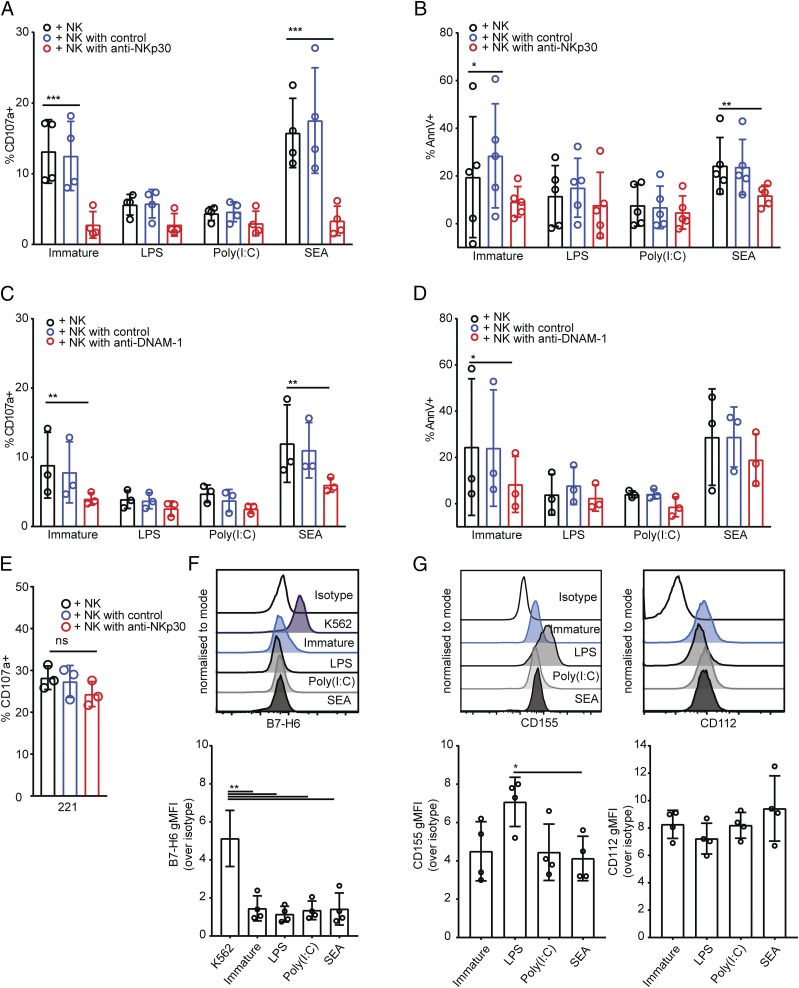
DNAM-1 and NKp30 contribute to lysis of DCs treated with SEA. (**A** and **B**) Percent of NK cells stained positive for CD107a (A) or stained with annexin V (B), after 5-h coculture with immature DCs or DCs treated for 24 h with LPS, poly(I:C), or SEA, where NK cells were preincubated for 1 h in culture medium alone (black bars), with an isotype-matched control Ab (blue bars), or NKp30 blocking Ab (red bars). Percent of annexin V–positive DCs cultured alone subtracted as background. (**C** and **D**) Percent of NK cells stained positive for CD107a (C) or stained with annexin V (D) after 5-h coculture with immature DCs or DCs treated for 24 h with LPS, poly(I:C), or SEA, where NK cells were preincubated for 1 h in culture medium alone (black bars) with an isotype-matched control Ab (blue bars) or DNAM-1 blocking Ab (red bars). Percentage of annexin V–positive DCs cultured alone subtracted as background. (**E**) Percentage of NK cells stained positive for CD107a after 5-h coculture with 721.221 target cells, NK cells preincubated for 1 h in culture medium alone (black bars) with an isotype-matched control Ab (blue bars) or NKp30 blocking Ab (red bars). (**F**) Expression of B7-H6 on the surface of immature DCs or DCs treated for 24 h with LPS, poly(I:C), or SEA. Histograms show representative overlays of live, CD11c^+^ CD14^−^ singlet DCs from representative donor (left) showing, from top to bottom, isotype-matched control staining, then mAb staining of K562 cells, immature DCs, or DCs treated with LPS, poly(I:C), or SEA. Graphs shows gMFI over isotype-matched control staining of DCs from four independent donors. (**G**) Expression of CD112 and CD155 on the surface of immature DCs or DCs treated for 24 h with LPS, poly(I:C), or SEA. Histograms show representative overlays of live CD11c^+^ CD14^−^ singlet DCs from representative donors (top) showing, from top to bottom, isotype-matched control staining, then mAb staining of immature DCs or DCs treated with LPS, poly(I:C), or SEA (as indicated). Graphs shows gMFI over isotype-matched control staining of DCs from four independent donors. Data points represent individual donors, bars show mean ± SD. (A–D) Analyzed by repeated measures two-way ANOVA with Dunnett multiple comparisons. (E) Analyzed by Kruskal–Wallis test with Dunn multiple comparisons. (F and G) Analyzed by repeated measures one way ANOVA with Tukey multiple comparisons. *p < 0.05, **p < 0.01, ***p < 0.001.

Both NKp30 and DNAM-1 have been previously shown to play a role in NK cell interactions with immature DCs (11, 19). To test whether these receptors are also required for the lysis of DCs treated with SEA, we pretreated NK cells with blocking Abs for NKp30 and DNAM-1 before introducing them to cocultures with immature DCs or DCs treated with LPS, poly(I:C), or SEA. Blocking NKp30 reduced the proportion of NK cells stained positive for CD107a after culture with immature DCs or DCs treated with SEA (Fig. 6A). In addition, staining of immature or Th2-polarizing DCs with annexin V following culture with NK cells was also significantly diminished in the presence of NKp30 blocking Ab (Fig. 6B). Thus, NKp30 is an important activating receptor involved in NK cell degranulation and subsequent apoptosis of immature or Th2-polarizing DCs.

Blocking DNAM-1 also prevented NK cell degranulation to immature DCs or Th2-polarizing DCs (Fig. 6C). However, although the proportion of immature DCs stained by annexin V following culture with NK cells was also significantly reduced by blocking DNAM-1 (Fig. 6D), there was little if any effect of blocking DNAM-1 in the fraction of Th2-polarizing DCs stained by annexin V (Fig. 6D). None of the blocking treatments had any effect on the proportion of NK cells stained positive for CD107a after culture with DCs treated with LPS or poly(I:C) (Fig. 6A, 6C). Blocking NKp30 or DNAM-1 had no effect on the proportion of DCs treated with LPS or poly(I:C) that showed annexin V staining (Fig. 6B, 6D). Confirming that blocking NKp30 does not abolish NK cell cytotoxicity in general, blocking NKp30 had no effect on NK cell degranulation to 721.221 target cells (Fig. 6E). Together, these results establish that NKp30 and DNAM-1 are important receptors for activation of NK cells triggered by immature DCs or Th2-polarizing DCs.

Because both NKp30 and DNAM-1 were important for lysis of immature DCs and DCs treated with SEA, we also set out to determine the expression of ligands for these receptors on the DC surface. B7-H6 is a ligand for NKp30 identified on the surface of tumor cells (61). Staining for B7-H6 was observed on the surface of K562 tumor cell lines but not on the surface of either immature or treated DCs (Fig. 6F). All DCs showed some expression of DNAM-1 ligands CD155 and CD112 (Fig. 6G). DCs treated with SEA showed lower CD155 expression compared with DCs treated with LPS. Overall, these data establish that NK cell receptors NKp30 and DNAM-1 play an important role in NK cell–mediated lysis of DCs treated with SEA, but the DC ligands important in this interaction are less well defined.

## Discussion

Cross-talk between NK cells and DCs influences the function of both cell types during inflammation (10–13). Mature DCs can enhance NK cell activity by providing cytokine signals such as IL-12, IL-15, and IL-18 (14–16). In turn, NK cells can facilitate the maturation of DCs via secretion of TNF-α and IFN-γ (20, 21) and can enhance DC antitumor responses (23, 24, 62). Although this interaction is well characterized in typical proinflammatory circumstances, the role of NK cell–DC cross-talk in the development of Th2 responses remains unexplored.

The helminth worm *S. mansoni* induces a strong Th2 inflammatory response (63), which relies on DCs for its initiation (34, 35). In this study, we used *S. mansoni* SEA to stimulate human monocyte-derived DCs to be capable of polarizing Th2 responses and assessed their interaction with autologous NK cells in vitro. DCs treated with SEA were similar to immature DCs in their lack of IL-12p70 production and low expression of CD86 and MHC molecules, as has been previously demonstrated (52, 53). However, DCs treated with both LPS and SEA showed decreased spreading and increased migration on fibronectin-coated surfaces in comparison with immature DCs (Fig. 2A–F). This provides a novel distinguishing feature for Th2-polarizing DCs in comparison with immature DCs.

NK cells conjugated with both immature DCs and DCs treated with SEA, resulting in movement of the NK cell MTOC toward the immune synapse and accumulation of integrin LFA-1 at the cell contact (Fig. 3A–E). This effect was not observed in NK cell conjugates with DCs treated with LPS or poly(I:C), which agrees with previous studies showing that the MTOC and perforin granules are not polarized to the immune synapse between NK cells and Th1-polarizing mature DCs (26). In cocultures between autologous NK cells and either immature DCs or DCs treated with SEA, we observed an induction of NK cell expression of CD107a, a marker of degranulation (Fig. 4A, 4B). We also identified an increase in the proportion of annexin V–stained apoptotic immature DCs or DCs treated with SEA after their culture with NK cells (Fig. 4C, 4D). In addition, DCs treated with glycoprotein omega-1, a known molecular component of SEA, similarly polarized Th2 responses and triggered degranulation of autologous NK cells (Fig. 4I, 4J). Imaging NK cell–DC interactions in a bespoke microwell system allowed tracking of individual cell contacts over time, further establishing that DCs treated with SEA were lysed by autologous NK cells (Fig. 5B).

Overall, in agreement with earlier research, we found that NK cells are capable of lysing autologous immature DCs (11). However, previous reports have suggested that as DCs mature and become able to activate T cells, they gain resistance to NK cell–mediated lysis. Our data reveal that this is not quite correct and that the type of T cell responses that DCs stimulate is critical to consider in this context. If DCs mature to be able to polarize Th1-type responses, then indeed they gain resistance to NK cell–mediated lysis. However, DCs that polarize a Th2 response can be readily lysed by autologous NK cells, at least in the specific case examined in this study of DCs stimulated by SEA.

To eliminate the possibility that NK cells were simply lysing nonactivated DCs remaining within the cultures of DCs treated with SEA, we treated DCs with fluorescently labeled SEA and observed their interactions with NK cells in microwells. NK cells preferentially lysed DCs that had taken up SEA (Fig. 5F, 5G). Taken together these data imply that NK cell killing of DCs could influence the polarization of an immune response. Indeed, other evidence also points to NK cell cytotoxicity being important in shaping the polarization of inflammation (64). NK cells have also shown an ability to lyse M2 macrophages in vitro via NKp46 and DNAM-1, likely because of their reduced expression of HLA class I molecules (65). The low expression of HLA class I observed on the surface of DCs treated with SEA (Fig. 1C) may contribute to their vulnerability to NK cell killing.

Lysis of both immature DCs and DCs treated with SEA was abrogated by Ab-mediated blockade of NKp30 and DNAM-1 (Fig. 6A–D). NK cells have previously been shown to lyse autologous immature DCs via NKp30 and DNAM-1 (11, 19). Thus, lysis of DCs treated with SEA appears to be triggered by similar molecular recognition. B7-H6 is an NKp30 ligand expressed on the surface of tumor cells, and expression can be induced on monocytes during inflammation (61, 66). However, in these studies, neither immature DCs nor DCs treated with SEA express B7-H6 (Fig. 6F). All DCs tested showed expression of DNAM-1 ligands CD155 and CD112 (Fig. 6G), which have been shown to contribute to NK cell lysis of both immature DCs and even some mature DCs (19). However, CD155 also binds inhibitory receptor TIGIT, suppressing both DC cytokine production and NK cell cytotoxicity (67, 68). Therefore, the expression of CD155 may have opposing influences on NK cell function depending on concurrent receptor interactions, for example, through NKp30.

Interestingly, chronic viral infections such HIV and hepatitis B have been linked with decreased expression of NKp30 on the NK cell surface (69–71). Coinfection with these viruses and helminths such as *S. mansoni* are common in areas where these infections are endemic (72, 73). Thus, the data we report in this paper lead us to speculate that alterations in NKp30 function on NK cells during these viral infections could impact NK cell editing of Th2-polarizing DCs and, in turn, impact host defense against helminths.

It remains to be tested whether NK cells lyse DCs stimulated in other Th2-polarizing conditions. Diminished NK cell number and function has also been associated with more severe asthma pathology (42); thus, it is possible that if the observations in this study extend to other Th2-polarizing circumstances outside stimulation with schistosome Ags, a reduction in NK cells capable of killing Th2-polarizing DCs could enhance pathogenic Th2 inflammation and disease.

Other important questions arising from this study are where and when NK cell lysis of Th2-polarizing DCs occurs in vivo. Multiple intravital imaging studies have shown that NK cells and DCs take part in both transient and long-lived interactions in lymph nodes (16, 27, 74, 75). In contrast, NK cells have also been proposed to lyse immature DCs at sites of inflammation (11), and NK cell–DC cross-talk can occur both in lungs during infection (76) and within tumors (23). This could suggest that NK cell lysis of DCs that have been stimulated by schistosome eggs to polarize Th2 responses may occur either in lymph nodes or in tissues proximal to deposited eggs. Determining the location of these interactions is an important next goal for understanding them in context.

Overall, we report that NK cells lyse schistosome egg–stimulated Th2-polarizing DCs via activating receptors NKp30 and DNAM-1. The importance of this in vivo remains to be established, but these observations lead us to speculate that NK cell editing of DCs can have an important influence on the polarization of immune responses.

## Supplementary Material

Data Supplement
