# Supplementary material for: Human NK Cells Lyse Th2-Polarizing Dendritic Cells via NKp30 and DNAM-1
Source: J Immunol. 2018 Aug 17;201(7):2028–41. doi: 10.4049/jimmunol.1800475 (PMC6139540; doi:10.4049/jimmunol.1800475)
Supplement: Data Supplement [file JI_1800475.zip › JI_1800475_Supplemental_Figure_1.pdf]

## Figure S1

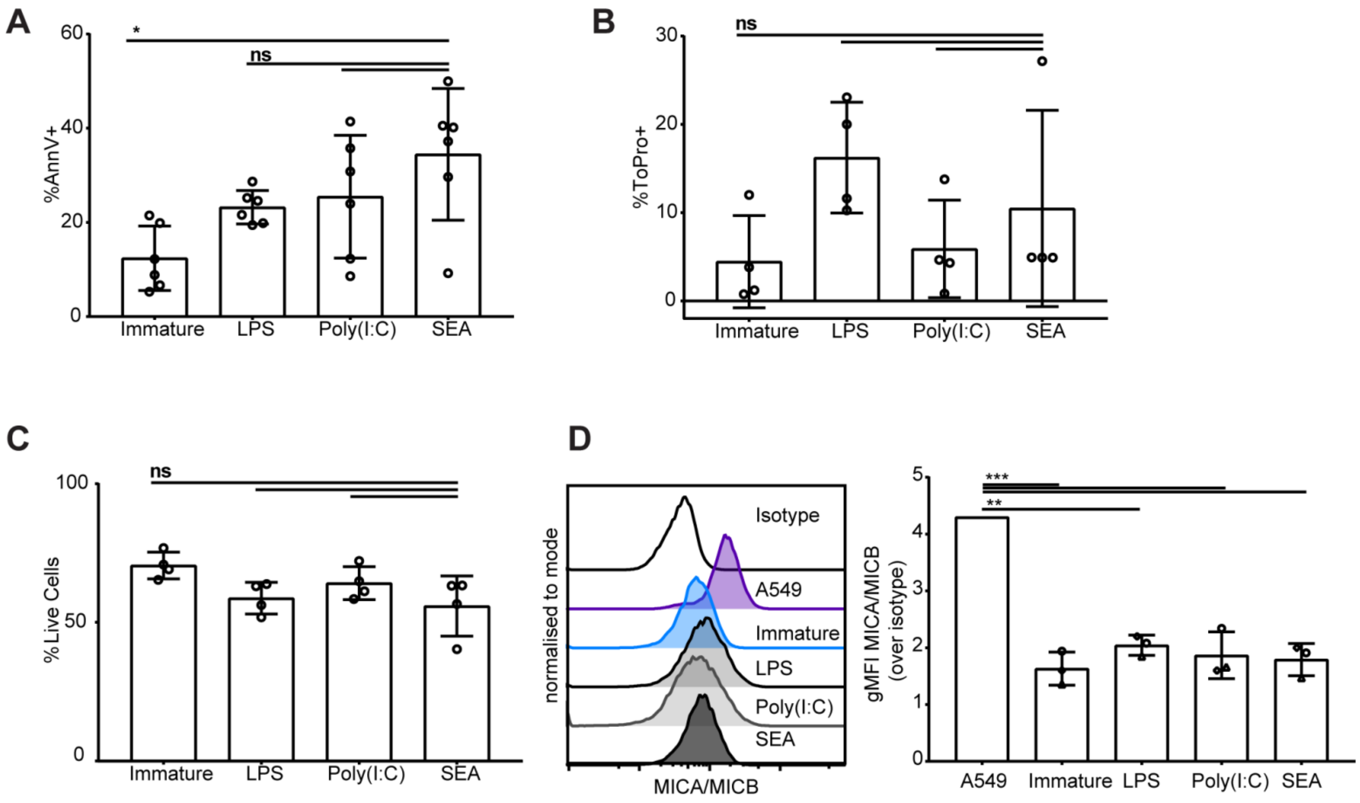


**Figure S1. DCs treated with SEA have similar viability to DCs treated with LPS or Poly(I:C). (A)** The proportion of immature DCs or DCs treated for 24 h with LPS, Poly(I:C) or SEA stained positive for annexin V after being washed and cultured alone for 5 h. **(B)** The proportion of immature DCs or DCs treated for 24 h with LPS, Poly(I:C) or SEA stained with cell death marker ToPro 3 Iodide after being washed and cultured alone for 5 h. **(C)** The proportion of live immature DCs or DCs treated for 24 h with LPS, Poly(I:C) or SEA as measured by staining with fixable viability dye. **(D)** Expression of MICA/MICB on the surface of immature DCs or DCs treated for 24 h with LPS, Poly(I:C) or SEA. Histograms show representative overlays of live, CD11c+ CD14- singlet DCs from representative donors (left), from top to bottom showing isotype matched control, then mAb staining of A549 tumour cells, immature DCs or DCs treated with LPS, Poly(I:C) or SEA respectively. Graphs show gMFI isotype matched control of DCs from 3 independent donors. In all plots shapes represent data points from individual donors and bars show mean (± standard deviation) of 3-6 independent donors. $p<0.05$, ** $p<0.01$, ***$p<0.001$; (A-C) analysed by Kruskal wallis test with Dunn's multiple comparisons. (D) Analysed by repeated measures one way ANOVA with Tukey's multiple comparisons.
